# Supplementary material for: Ceria Quantum Dot Filler-Modified Polymer Electrolytes for Three-Dimensional-Printed Sodium Solid-State Batteries
Source: Polymers (Basel). 2024 Jun 14;16(12):1707. doi: 10.3390/polym16121707 (PMC11207215; doi:10.3390/polym16121707)
Supplement: Supplementary file 1 [file polymers-16-01707-s001.zip › polymers-3057789-supplementary.pdf]

Supporting Information

Ceria Quantum Dots Fillers Modified Polymer Electrolytes for 3D-Printed Sodium Solid-State batteries

Yi Zhang, Haoran Zheng, Honggeng Ding, Khan Abdul Jabbar, Ling Gao* and Guowei, Zhao*

College of Chemistry and Chemical Engineering, Huanggang Normal University, Huanggang 438000, China; legend10086@163.com(Y.Z.); 1821928258@qq.com (H.Z.); xxxxx@xxx(H.D.), niaoba220284@163.com (H.L.); khan@hgnu.edu.cn (K.J.); gaoling@hgnu.edu.cn(G.L.); zhaoguowei@hgnu.edu.cn (G.Zhao)

* Correspondences: gaoling@hgnu.edu.cn; zhaoguowei@hgnu.edu.cn

ORCID:

Y.Y.: 0000-0003-2423-2175

K. J.: 0000-0002-8436-3831

L.G.: 0000-0002-3171-591X

G.Z.: 0000-0002-4344-3923

Figures and Tables

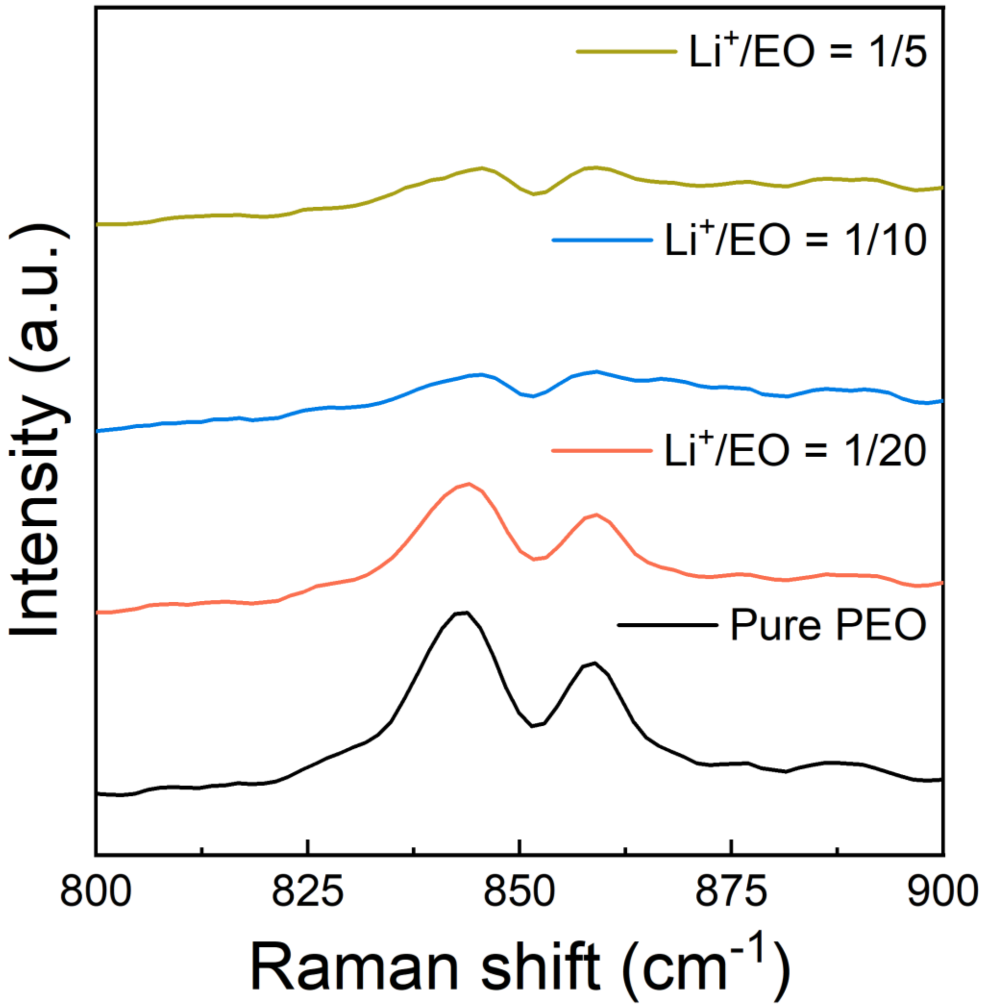

Figure S1. Raman spectra of SPE with different Na^{+}/EO molar ratio.

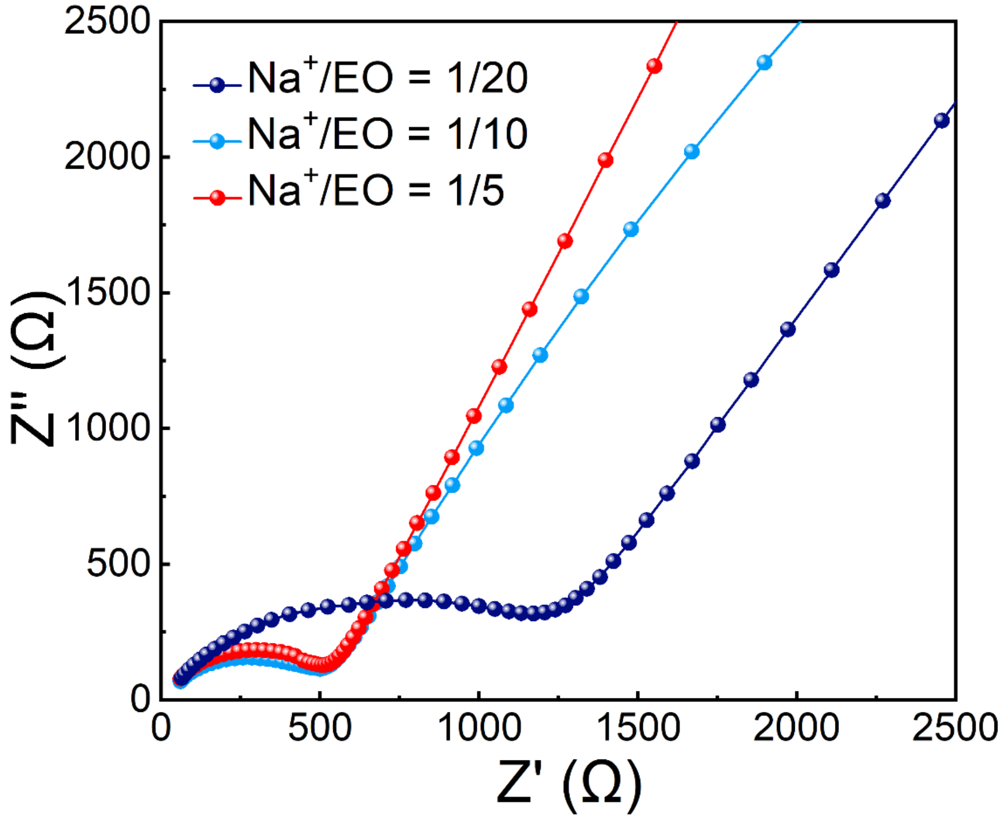

Figure S2. EIS test of SPE with different Na^+/EO molar ratio.

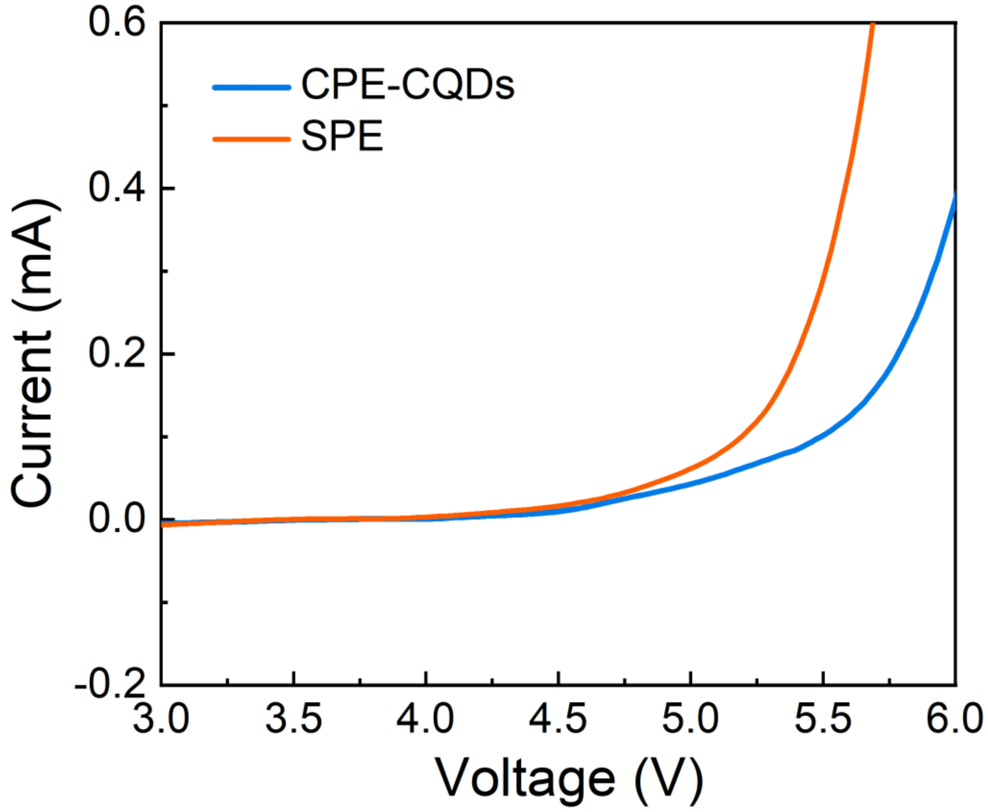

Figure S3. LSV test of SPE and CPE-CQDs membrane.

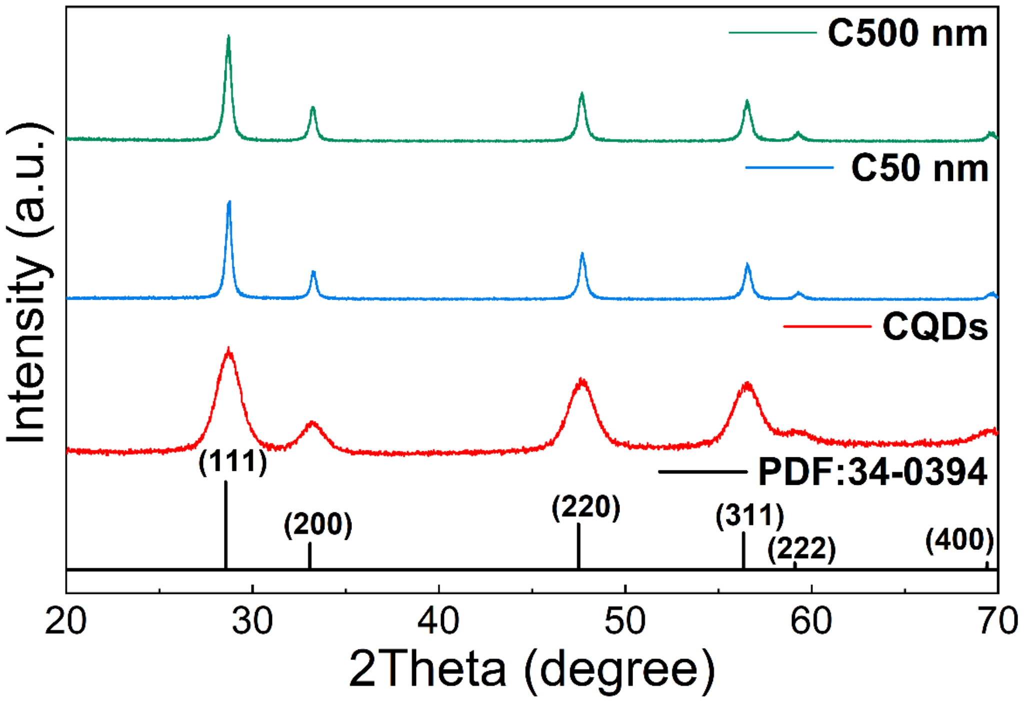

Figure S4. XRD patterns of the CQDs, C50 and C500 samples.

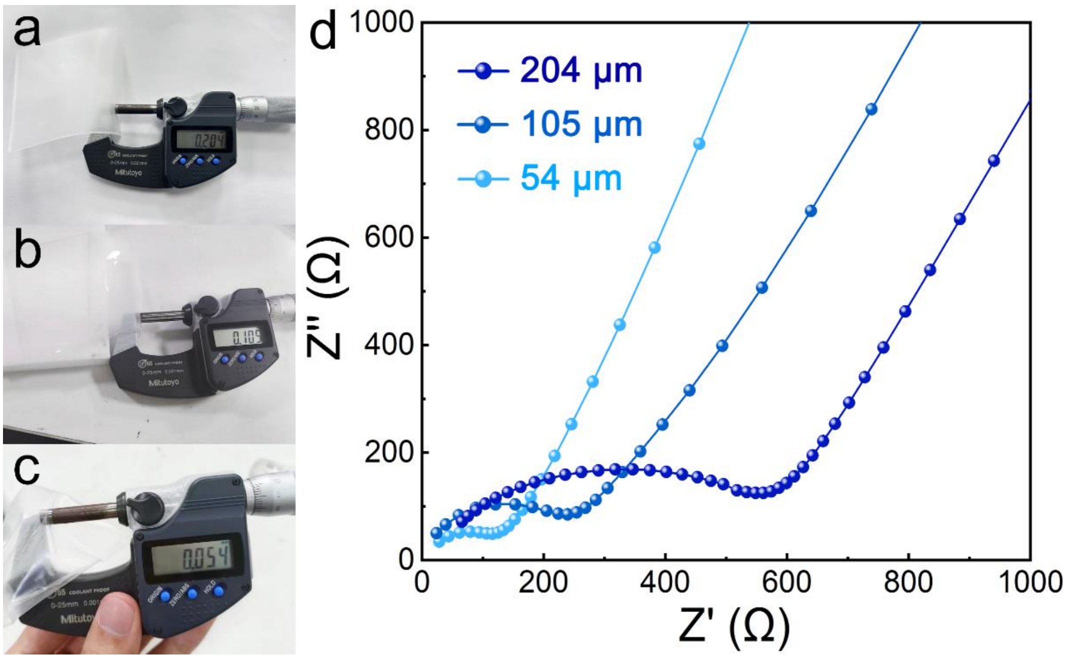

Figure S5. (a-c) The printed CPE-CQDs membranes with different thickness. (d) EIS test of the CPE-CQDs membranes with different thickness.

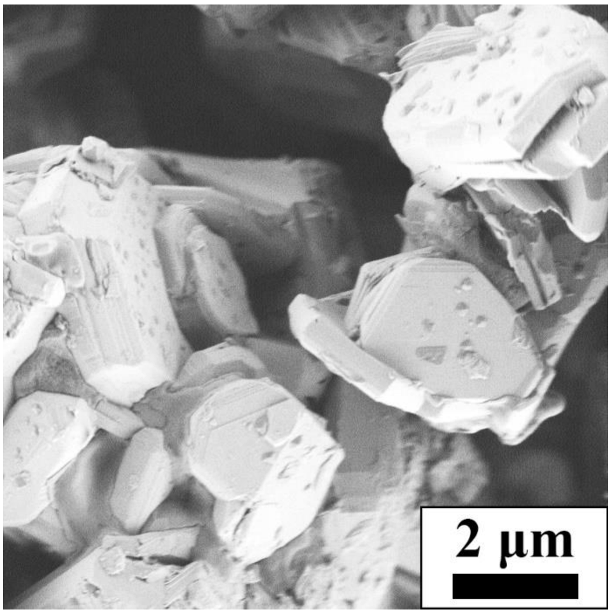

Figure S6. SEM image of NNM sample.

Table S1. Crystallographic parameters of ceria and NNM refined by the Rietveld methode.

Refine Result	a	b	c	V	R_p	R_{wp}
**ceria**	**5.423602 Å**	**5.423602Å**	**5.423602 Å**	**159.538 Å^3**	**3.56%**	**4.18%**
**NNM**	**2.890553 Å**	**2.890553 Å**	**11.164302 Å**	**80.793 Å^3**	**3.35%**	**4.95%**

Table S2. The comparation of sodium solid state battery.

Cathode	Electrolyte	C-Rate	Cycle number	Residual Capacity (mAh/g)	Retention (%)	Mass loading mg/cm^2	Size	Test °C	ref
**$Na_{2/3}Ni_{1/3}Mn_{2/3}O_2$**	PEO/NaFSI	0.1 C	100	140	99.91	2	Coin cell	20	1
**$Na_{2/3}Ni_{1/3}Mn_{2/3}O_2$**	P(EO/MEEGE)	1/30 C	30	69	93	2.1	Coin cell	60	2
**$Na_3V_2(PO4)_3$**	NaFSI/PEO/Al_2O_3	0.25 mA/cm^2	2000	84	93	3	Coin cell	80	3
**$Na_3V_2(PO4)_3$**	$NaClO_4$/PEO/Al_2O_3	2 C	1000	84	87.5	2.2	Coin cell	80	4
**$Na_3V_2(PO4)_3$**	$NaClO_4$/PMA/α-Al_2O_3	0.5	350	80	94.1	Not mention	Coin cell	70	5
**$Na_3V_2(PO4)_3$**	$NaClO_4$/PEO/CQDs	1	200	50	45.1	2	Coin cell	60	6
**$Na_3V_2(PO4)_3$**	NaTFSI/PEO/$Na_{3.4}Zr_{1.8}Mg_{0.2}Si_2PO_{12}$	0.1	120	100	99	2.5	Coin cell	80	7
**$Na_2MnFe(CN)_6$**	$NaClO_4$/PEO/$Na_3Zr_2Si_2PO_{12}$	0.5	300	124	72	3	Coin cell	60	8
**$Na_{2/3}Ni_{1/3}Mn_{2/3}O_2$**	PEO/NaTFSI/Ceria	0.2 C	200	74	92	1~1.2	4 x 4 cm	30	This work

(1) Roscher, D.; Kim, Y.; Stepien, D.; Zarrabeitia, M.; Passerini, S. Solvent‐free Ternary Polymer Electrolytes with High Ionic Conductivity for Stable Sodium‐based Batteries at Room Temperature. *Batteries & Supercaps* **2023,** *6* (9), e202300092.

(2) Tatara, R.; Suzuki, H.; Hamada, M.; Kubota, K.; Kumakura, S.; Komaba, S. Application of P2-Na2/3Ni1/3Mn2/3O2 Electrode to All-Solid-State 3 V Sodium(-Ion) Polymer Batteries. *Journal of Physical Chemistry C* **2022,** *126* (48), 20226-20234.

(3) Liu, L.; Qi, X.; Yin, S.; Zhang, Q.; Liu, X.; Suo, L.; Li, H.; Chen, L.; Hu, Y.-S. In Situ Formation of a Stable Interface in Solid-State Batteries. *ACS Energy Lett.* **2019,** *4* (7), 1650-1657.

(4) Gao, R.; Tan, R.; Han, L.; Zhao, Y.; Wang, Z.; Yang, L.; Pan, F. Nanofiber networks of Na3V2(PO4)3 as a cathode material for high performance all-solid-state sodium-ion batteries. *J. Mater. Chem. A* **2017,** *5* (11), 5273-5277.

(5) Zhang, X.; Wang, X.; Liu, S.; Tao, Z.; Chen, J. A novel PMA/PEG-based composite polymer electrolyte for all-solid-state sodium ion batteries. *Nano Research* **2018,** *11* (12), 6244-6251.

(6) Ma, C.; Dai, K.; Hou, H.; Ji, X.; Chen, L.; Ivey, D. G.; Wei, W. High Ion-Conducting Solid-State Composite Electrolytes with Carbon Quantum Dot Nanofillers. *Adv. Sci.* **2018,** *5* (5), 1700996.

(7) Zhang, Z.; Zhang, Q.; Ren, C.; Luo, F.; Ma, Q.; Hu, Y.-S.; Zhou, Z.; Li, H.; Huang, X.; Chen, L. A ceramic/polymer composite solid electrolyte for sodium batteries. *J. Mater. Chem. A* **2016,** *4* (41), 15823-15828.

(8) Yu, X.; Xue, L.; Goodenough, J. B.; Manthiram, A. A High-Performance All-Solid-State Sodium Battery with a Poly(ethylene oxide)–Na3Zr2Si2PO12 Composite Electrolyte. *ACS Mater. Lett.* **2019,** *1* (1), 132-138.
